# Long-term exposure to particulate matter from road traffic and residential heating and mortality: a multi-cohort study in Sweden

**DOI:** 10.1038/s41598-026-37471-5

**Published:** 2026-02-27

**Authors:** Leo Stockfelt, Bertil Forsberg, Eva M. Andersson, Niklas Andersson, Gerd Sallsten, David Segersson, Kristina Eneroth, Lars Gidhagen, Peter Molnar, Mikael Ögren, Patrik Wennberg, Annika Rosengren, Debora Rizzuto, Karin Leander, Patrik K. E. Magnusson, Lars Barregard, Tom Bellander, Göran Pershagen, Petter L. S. Ljungman, Johan Nilsson Sommar

**Affiliations:** 1 Occupational and Environmental Medicine, School of Public Health and Community Medicine, Institute of Medicine, Sahlgrenska Academy, University of Gothenburg, Gothenburg, Sweden; 2 Occupational and Environmental Medicine, Sahlgrenska University Hospital, Gothenburg, Sweden; 3 Department of Epidemiology and Global Health, Umeå University, Umeå, Sweden; 4 Institute of Environmental Medicine, Karolinska Institutet, Stockholm, Sweden; 5 Swedish Meteorological and Hydrological Institute, Norrköping, Sweden; 6 SLB-analys, Environment and Health Administration, Stockholm, Sweden; 7 Department of Public Health and Clinical Medicine, Family Medicine, Umeå University, Umeå, Sweden; 8 Department of Molecular and Clinical Medicine, Institute of Medicine, University of Gothenburg, Sahlgrenska University Hospital, Gothenburg, Sweden; 9 Region Västra Götaland, Sahlgrenska University Hospital, Gothenburg, Sweden; 10 Ageing Research Center, Department of Neurobiology, Care Sciences and Society, Karolinska Institutet and Stockholm University, Stockholm, Sweden; 11 Department of Medical Epidemiology and Biostatistics, Karolinska Institutet, Stockholm, Sweden; 12 Department of Cardiology, Danderyd Hospital, Stockholm, Sweden

**Keywords:** Air pollution, Particulate matter, Natural mortality, Cardiovascular mortality, Source-apportionment, Cardiology, Diseases, Environmental sciences, Risk factors

## Abstract

Exposure to particulate air pollution increases total natural and cardiovascular mortality. However, it is less clear which types and sources of particles are the most harmful. We analyzed associations between long-term exposure to source-specific locally emitted particles and total natural and cardiovascular mortality in Swedish cohorts. Using high-resolution dispersion models of particles from different sources, and address registries, we assigned annual individual residential mean concentrations to population-based cohorts in Gothenburg, Stockholm and Umeå 1990–2011. Time and cause of death were assigned from registries. Associations between long-term mean lagged exposures and mortality were estimated using Cox regression models adjusted for possible confounders, and meta-analyzed. 7344 natural deaths, including 2755 cardiovascular deaths, occurred among the 68,679 participants. Exposure levels were moderate but generally above the WHO 2021 guidelines. We observed positive associations with natural mortality for the last five years of exposure to road traffic exhaust particles (HR 1.02, 95% CI 1.00-1.04, per IQR, and 1.10, 95% CI 1.00-1.22, per 1 µg/m^3^), and road wear particles (HR 1.02, 95% CI 1.00-1.04, per IQR, and HR 1.02, 95% CI 1.00-1.03, per 1 µg/m^3^), but not for particles from residential heating. Adjustment for road traffic noise, or particles from residential heating, did not substantially affect the results for traffic-related particles. For cardiovascular mortality, associations with particles from both sources were positive but not statistically significant. Natural mortality was associated with local emissions of traffic-related particles in a multi-cohort study at moderate exposure-levels, lending some support for further efforts to reduce traffic emissions.

**Supplementary Information:**

The online version contains supplementary material available at 10.1038/s41598-026-37471-5.

## Introduction

Long-term exposure to ambient particulate matter (PM) air pollution is associated with increased mortality^1^. The global health burden is vast. The Global Burden of Disease Study in 2024 ranked exposure to PM air pollution as the specific risk factor which contributed the most to the global disease burden^2^. Background concentrations of ambient PM_2.5_ (particles with an aerodynamic diameter ≤ 2.5 μm) were estimated to be responsible for 4.7 million excess deaths globally in 2021, mainly due to an increase in cardiovascular disease (CVD) mortality. The economic cost has been estimated to be two trillion dollars annually^3^.

Burden of disease estimates for ambient air pollution are based primarily on long-term exposure to total PM_2.5_ concentration because this is the exposure metric for which the epidemiological evidence is most extensive, and the burden of proof is strongest. Air pollution is, however, a complex mixture of gases and particles from various sources, and health effects are likely to vary depending on size-dependent pulmonary deposition patterns as well as differences in toxicity between different components^4^. It is important to identify which types and sources of air pollution are the main contributors to adverse health effects to enable policy, guidelines and technical development to target the most harmful sources and the most effective preventive measures.

A growing number of epidemiological studies have investigated health effects of source-specific PM and of different components of air pollution. There are some indications that PM from coal burning and road traffic exhaust incur greater health effects than PM from other sources^5,6^. Overall, the evidence is still heterogeneous and inconclusive regarding possible differences in effects on mortality and other health effects. More research about which components and sources of PM that are most responsible for negative health effects have thus been repeatedly called for^5–8^.

Two major sources of primary PM globally and in Europe are emissions from road traffic and residential heating^9^, both highly relevant for population exposure. Emissions from road traffic and residential heating are also highly modifiable through technological substitutions and development, e.g. electrification of the vehicle fleet and wood stove combustion exchanges, and therefore possible to affect through targeted incentives and legislation. In this study we used high-resolution dispersion models separating source contributions to study associations between long-term exposure to locally emitted PM air pollution from road traffic (exhaust and road wear) and residential heating with total natural and CVD mortality, in population-based cohorts in three Swedish cities. The air pollution levels were comparatively low, making the setting relevant for informing guidelines and long-term preventive action.

## Materials and methods

### Study population

#### Cohorts

The study cohorts have been described previously in more detail^10,11^. Briefly, we included one cohort consisting of participants in the Västerbotten intervention program (VIP) living in Umeå municipality^12^, four cohorts from the Stockholm region (the Swedish National Study of Aging and Care in Kungsholmen (SNAC-K)^13^, the Stockholm Screening Across the Lifespan Twin Study (SALT)^14^, the Stockholm cohort of 60-year-olds (60YO)^15^, and the Stockholm Diabetes Prevention Program (SDPP)^16^) that have previously been combined and harmonized into one cohort (the Cardiovascular Effects of Air pollution and Noise Study (CEANS)^17^ and two from the Gothenburg region (the Primary Prevention Study (PPS) cohort^18^, and the Gothenburg subcohort of the WHO “Multinational Monitoring of Trends and Determinants in Cardiovascular Diseases” (GOT-MONICA, or GM) project)^19^. All the cohorts are population-based prospective cohorts in Sweden recruited to study cardiovascular and metabolic diseases or ageing, and mostly middle-aged at recruitment 1971–2013. All participants in the cohorts have given informed consent, and the study was reviewed and approved by Ethical Review Boards of Gothenburg (references T800-08 and T547-13), Stockholm (reference 2018/2064-32), and Umeå (reference 2015/16–31Ö).

#### Individual covariates

Baseline data on lifestyle factors (self-reported smoking, physical activity, alcohol consumption), demographic factors (age, sex) and socioeconomic factors (occupational status and class, education level, marital status) were collected at recruitment and are summarized in Table 1.

**Table 1:** Selected characteristics at enrollment for the participants in the four cohorts, and the numbers of total natural and cardiovascular deaths in each cohort during the study period. For some variables, notably marriage status and education level in GM and alcohol consumption in VIP, a large proportion of the answers were missing due to the questions not being included certain years. Na = not available.

Cohort		GM	PPS	CEANS	VIP
Participants, n		4,500	5,850	22,314	42,580
Recruitment year		1985,-90,-95	1970-74	1992–2004	1992–2014
Enrolment age, median (range)		46 (25–66)	51 (47–56)	56 (35–104)	40 (40–50)
Women, %		52	0	58	52
Smoking status,%	Current smoker	29	39	22	19
	Former smoker	na	33	36	30
	Never-smoker	65^a^	27	40	49
Leisure time physical activity^e^, %	Sedentary	18	24	61	36
	Moderate	62	59	26	42
	Intermediate & vigorous	18	17	7.6	22
Alcohol consumption^e^, %	Daily	na	na	6.7	2
	Weekly	na	na	55	16
	Seldom	na	na	31	42
	Never	na	na	5.6	1
Married/cohabitating, %	No	21	14	29	23
	Yes	47	86	70	77
Education level, %	Primary school or less	13	na	30	30
	Up to secondary school	32^b^	na	36	30
	University degree	20	na	31	40
Occupation, %	Gainfully employed	na	na	66	85
	Unemployed	na	na	5.8	6
	Retired	na	na	27	4
	Missing data	na	na	1.3	4
Occupational class, %	Blue collar	na	48	27	na
	Low/intermediate white-collar and self-employed	na	23	51	na
	High-rank white-collar and professionals	na	30	18	na
Mean area income (SEK)		154,780^c^	148,602^c^	303,910^d^	130,000^c^
Natural mortality, n	ICD9 001-779, ICD-10 A00-R99	532	3036	2420	1356
Cardiovascular mortality, n	ICD-9 400–440, ICD-10 I10-I70	183	1420	840	312

a) Includes non-smokers, b) Includes technical training, c) in 1994. d) in 2009. e) In CEANS the categories were defined as “Once a month or less/<1 h/week”, About once a month/~1 h/week”, and 3 times a week or more/>2 h/week. GM = GOT-MONICA, PPS= Primary Prevention Study, CEANS= Cardiovascular Effects of Air pollution and Noise Study, VIP= Västerbotten Intervention Program, SEK=Swedish crowns, ICD=International Classification of Diseases, na = not available in this cohort.

#### Mortality outcomes

We retrieved data on total natural and cause-specific mortality from the Cause of Death registry of the Swedish National Board of Health and Welfare and linked to participants of the four cohorts through their unique Swedish personal identification number. Natural mortality was defined as deaths where the primary cause was coded as International Classification of Diseases (ICD)-9 001-779 or ICD-10 A00-R99 (only excluding violent and accidental deaths) and cardiovascular (CVD) mortality as ICD-9 400–440 or ICD-10 I10-I70).

#### Address data and area-level socioeconomy

For all participants we retrieved annual residential address history through linkage based on their unique Swedish personal identification numbers with mandatory records of residential addresses at Statistics Sweden and the Swedish Tax Agency from 1 January 1990 and to 31 December 2011. We geocoded all addresses through matching against the Swedish Mapping Cadastral and Land Registration Authority databases. Area level socioeconomic status was estimated for each residence as the mean neighborhood individual income in persons of working age by Small Areas for Market Statistics (SAMS) from Statistics Sweden, using data from 1994 for Gothenburg and Umeå and from 2009 for Stockholm.

### Exposure assessment

Data on exposure assessment has been both described extensively^20^ and summarized previously^10,11^. Briefly, we used Gaussian dispersion models included in the Airviro air quality management system^21^. These particular models make use of a diagnostic wind model based on the concept first described by Danard^22^, in which it is assumed that small scale winds can be seen as a local adaptation of large-scale winds. The model estimates air pollution concentrations 2 m above ground level and treats the buildings by using a roughness parameter. Annual mean concentrations were calculated based on simulated concentrations, either hour-by-hour (Gothenburg and Umeå) or using a representative climatology with 60 wind sectors and 6 stability classes (Stockholm), at a spatial resolution down to 35 × 35 m^2^. Emissions were taken from local and regional inventories for the years 1990, 2000 and 2011 in Gothenburg and Umeå, and the years 1990, 1995, 2000, 2005 and 2011 in Stockholm. Mean concentrations for intervening years were interpolated linearly, and for Gothenburg and Umeå corrected with a year-specific ventilation index calculated from air dispersion modeling over the two cities for each year from 1990 to 2011. Annual average regional background PM_2.5_ and PM_10_ concentrations were assumed to be the same within each city, since the spatial variation between regional background stations is low^23^. For Stockholm and Umeå long-range contributions were taken from regional background stations outside the cities, and in Gothenburg calculated by subtracting the modelled local contributions from monitored concentrations at an urban background station.

The local contributions of source-specific locally emitted particles with an aerodynamic diameter of ≤ 10 μm (PM_10_) and ≤ 2.5 μm (PM_2.5_) generated within of the most important source categories in urban settlements were modelled separately: Road traffic exhaust, non-exhaust road traffic emissions, residential heating, shipping, industry, and other activities (e.g. off-road machinery, agricultural sources). Particle emission factors for traffic exhaust were calculated using geographical data on the use of different vehicle types, speeds and driving conditions based on the Handbook on Emission Factors for Road Traffic version3.1^24^. The non-exhaust emissions from traffic consist mainly of road wear PM with minor contributions from brake and tire wear. The emission of non-exhaust PM increases with road traffic speed and the share of studded tires used during wintertime conditions. For small-scale residential heating in Umeå, a detailed inventory of individual stoves and boilers was used with data from chimney sweepers and interviews about amount of wood burning^20^. Residential heating emissions in Gothenburg and Stockholm were distributed top-down based on household energy consumption data from Statistics Sweden and distributed spatially with a resolution of 100 m x 100 m using proxy-data such as number of stoves or boilers in each municipality, living space of detached housing per km^2^, population density per 100 m^2^ and availability of district heating. Industrial and energy production facilities were in the model included as point sources, and emissions were retrieved from the Swedish national emission inventory^25^ or directly from the supervisory authorities. Where relevant, the emission inventories also included other sources such as off-road machinery and diffuse emissions related to agriculture. Diffuse emission, typically sources with varying or unknown coordinates, were mainly distributed using top-down methods at a coarser spatial resolution (around 1 km x 1 km), with slightly different methods in Stockholm compared to Gothenburg and Umeå. For Stockholm, diffuse emissions with unknown spatial distribution, e.g. off-road machinery, were excluded from the emission inventory, resulting in lower exposure from this source category. Emissions from shipping were also included using a method similar to Jalkanen et al.^26^. The emissions were described using a bottom-up approach including actual ship movements of all ships equipped with Automatic Identification System (AIS) transponders and ship properties acquired from international databases (Lloyds Register Fairplay and MARS database of the International Telecommunications Union). The annual average distribution of emissions was used in the modeling and introduced as grids with a resolution of 1 km x 1 km.

More detailed descriptions of the models and the emission inventories used, as well as an evaluation of model results against measurements, are given in^20^. There is also a discussion of the main characteristics of the simulated exposure of particulate matter, showing that emissions from road traffic and residential heating were the dominant local sources in the studied cities, with local shipping only contributing to exposure for the population living in direct vicinity of quays or fairways in harbor areas, and the contribution from other diffuse sources was relatively small with variations in modelling methodology between cities. In the following text we denote PM_2.5_ particles from road traffic exhaust as “PM_exhaust_”, PM_10_ particles from road traffic non-exhaust as “PM_wear_”, these exposures combined as “PM_traffic_”, and PM_2.5_ from residential heating as “PM_residential heating_”.

Residential exposure to road traffic noise was assigned in the Gothenburg and Stockholm cohorts to control for possible confounding by co-exposure. It was estimated as the equivalent A-weighted sound pressure level on the most exposed façade, using the Nordic method for road traffic noise prediction^27^, with a simplified approach for receiver positions in dense urban areas^28^. For Gothenburg residential noise was calculated each year, while for Stockholm noise was estimated every 5 years and intervening years interpolated linearly.

All participants were assigned annual mean residential exposures for every year of the study period 1990–2011, for which they had a geocoded address within the modelled areas.

### Statistical methods

We used a Cox proportional hazards model to estimate the association between air pollutant exposures and total natural, and CVD mortality in each cohort, respectively, using the same covariate models as described previously^11^. The crude model included only age as the time-scale, calendar year, sex, and in CEANS an indicator for subcohort. The main covariate model additionally adjusted for baseline information on probable individual- and area-level confounders: smoking status (current/former/never), alcohol consumption (only in Stockholm and Umeå: Daily/weekly/seldom/never), physical activity (in Gothenburg and Umeå: Sedentary/moderate/intermediate & vigorous, in Stockholm: Once a month or less or < 1 h per week/about once a week or approximately 1 h per week/3 times a week or more or > 2 h per week), marital status (single/married or living with partner/no answer), socioeconomic index by occupational class (Blue-collar/low and intermediate white-collar and self-employed/high-level white-collar and self-employed/professionals with academic degrees/no answer), level of education (Primary school or less/up to secondary school or equivalent/university degree or more/no answer), occupational status (gainfully employed/unemployed/retired/no answer), and neighborhood individual income in persons of working age. Because certain covariates were not collected in all cohorts (alcohol consumption and level of education in GM and PPS, occupational class in VIP), cohort-specific models were adjusted for the covariates available within each specific cohort. These harmonized models were then pooled in the random-effects meta-analysis, which reduces bias arising from differences in covariate availability. Since the primary biological pathways between air pollution exposure and natural and CVD mortality are inflammation and cardiometabolic dysregulation^4^, cardiometabolic risk factors such as blood pressure, diabetes and body mass index (BMI) were considered likely mediators on the causal pathway and not adjusted for, to avoid overadjustment.

Associations with mortality outcomes were analyzed separately for PM_wear_ and PM_exhaust_ as well as combined as PM_traffic_, and for PM_residential heating_. In two-pollutant models, both PM_exhaust_ and PM_residential heating_ were included, as well as both PM and road traffic noise. The main exposure time-window was defined as the moving average exposure of the last five years (lag 1–5). Participants were excluded from analyses if exposure could not be assigned for at least 80% of the years within the exposure window (i.e., four out of five years). For example, in Gothenburg, the first year with at least 80% exposure data was 1994, based on the mean over the four years 1990–1993, making the study period 1994–2011. In secondary analyses, we also examined the average exposure 6–10 years prior (lag 6–10). Participants were censored at death from causes other than the outcome in question, at the end of the study period, or upon permanent emigration from the study area.

Hazard ratios (HR) and 95% confidence intervals (CIs) were expressed per interquartile range (IQR, defined as average interquartile ranges across the study areas), and for the main analyses also per fixed increment of 1 µg/m^3^, and per 10 dB L_DEN_ (day-evening-night level) for road traffic noise. Estimates from cohort-specific analyses were pooled using a random-effects meta-analysis^29^. Heterogeneity between cohort estimates was assessed by the I^2^ statistic, and statistical deviations from homogeneity were tested with a Chi-square test based on Cochran’s Q statistic.

## Results

### Participant characteristics

The total number of participants included was 68,679, of which 7344 died of any natural cause and 2755 of cardiovascular causes during the study period. The numbers of deaths due to each cause in each cohort, as well as background covariates, are presented in Table 1. The participants were on average the oldest at study start in the PPS cohort, and mortality rate during the study period was consequently the highest in this cohort, while the VIP cohort participants were the youngest and had the lowest mortality rate. The PPS cohort included only men, while the other three cohorts had a slightly larger proportion of women. Smoking was most prevalent in the PPS cohort, reflecting the high smoking rates in the 1970s. Regarding leisure time physical activity, sedentary lifestyle seemed the most common in the CEANS cohort. Alcohol consumption was more frequent in CEANS compared with VIP, and having attained a university education was more common in CEANS and VIP compared to PPS and GM, likely reflecting secular trends. Blue-collar occupations were more common in the Gothenburg PPS cohort than in CEANS. A larger proportion of individuals were gainfully employed in VIP compared with CEANS.

### Exposure characteristics

**Fig. 1:**
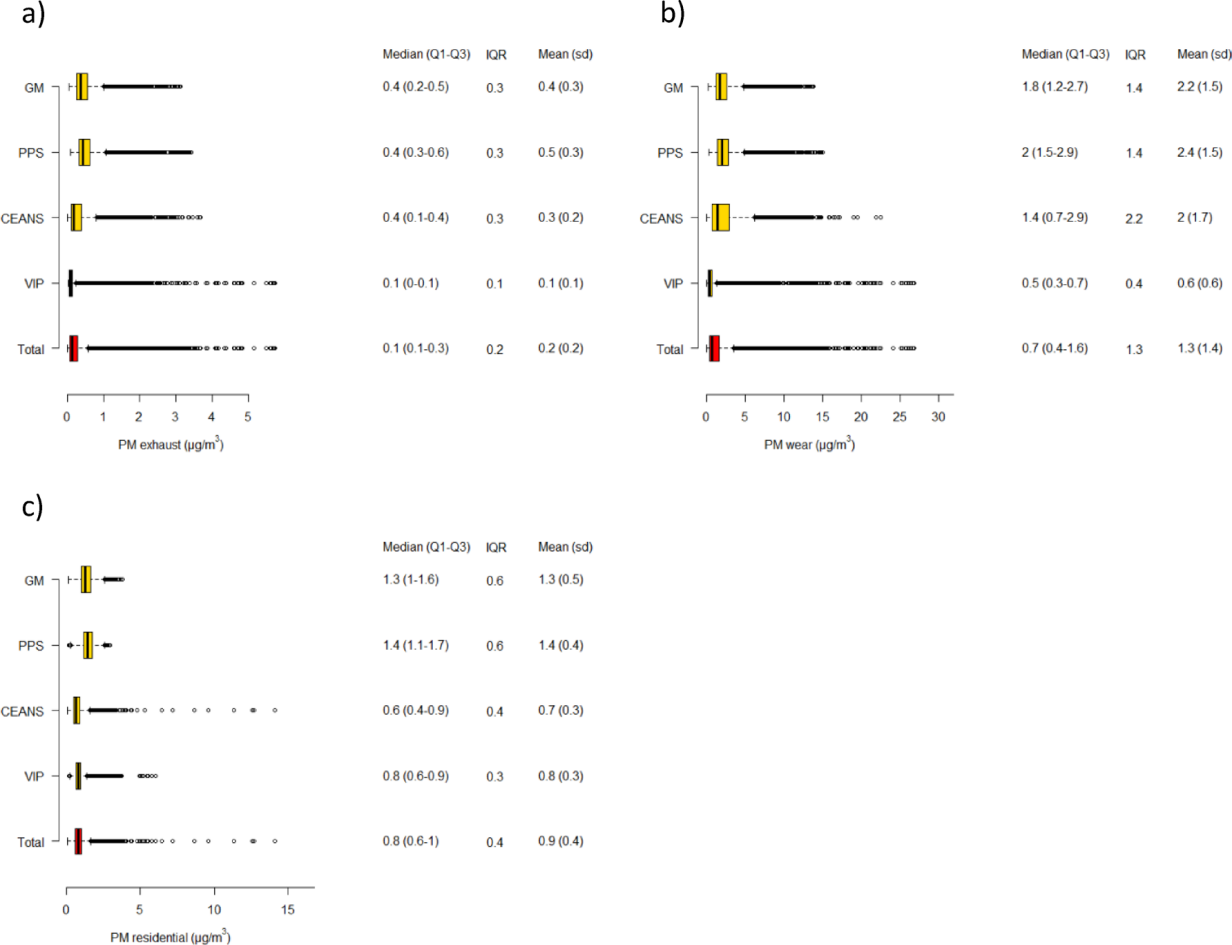
(**a**–**c**) Boxplots of (**a**) PM_exhaust_, (**b**) PM_wear_ and (**c**) PM_residential heating_ during the study period 1990–2011 in total and separate for the four cohorts. Tabulated values are quartiles (Q1 = quartile 1, Q3 = quartile 3), interquartile range (IQR), means and standard deviations (sd). GM = GOT-MONICA, PPS= Primary Prevention Study, CEANS= Cardiovascular Effects of Air pollution and Noise Study, VIP= Västerbotten Intervention Program.

**Fig. 2:**
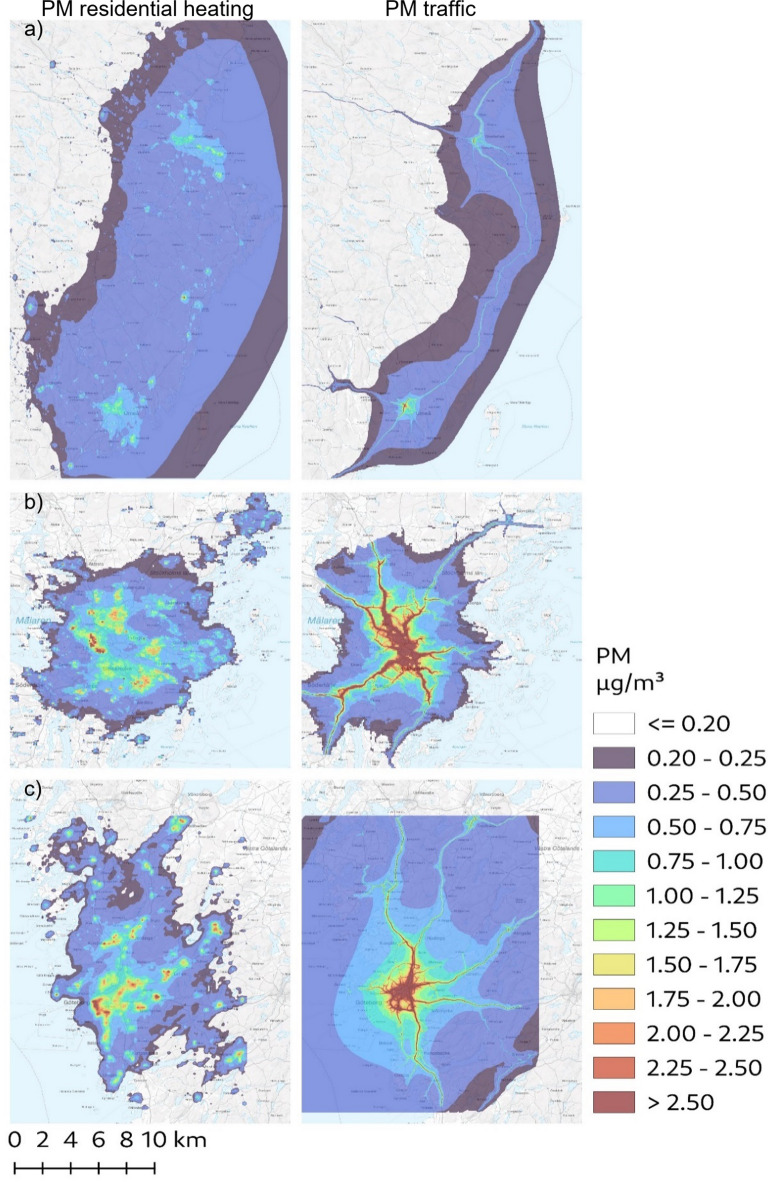
(**a**–**c**) The distribution of locally emitted particulate matter (PM) from residential heating (left) and road traffic (right, road wear and exhaust combined) in the study areas in and around (**a**) Umeå, (**b**) Stockholm, and (**c**) Gothenburg in the year 2011. The map has been produced with QGIS v3.16 and the background map is public domain (CC0) provided by the Swedish national mapping, cadastral and land registration authority.

The mean exposures to local emissions of PM_exhaust_, PM_wear_ and consequently combined PM_traffic_ were higher in the Stockholm and Gothenburg cohorts compared to Umeå (Fig. 1a–c, suppl Fig. 1). PM_residential heating_ was also the highest in the Gothenburg cohorts, with lower and approximately equivalent levels in Stockholm and Umeå. The combined interquartile ranges were 0.2 µg/m^3^ for PM_exhaust_, 1.3 µg/m^3^ for PM_wear_, 1.5 µg/m^3^ for PM_traffic_ and 0.4 µg/m^3^ for PM_residential heating_. Lag 1–5 year concentrations were slightly lower than lag 6–10 years due to a decreasing trend over time for most pollutants (data not shown). The spatial distribution of PM_traffic_ and PM_residential heating_ in the year 2011 is illustrated in Fig. 2a–c. The pattern was similar for PM_exhaust_ and PM_wear_, which were highly correlated with each other (*r* = 0.94), but the levels of PM_wear_ approximately six times higher than PM_exhaust_ in terms of µg/m^3^. The correlations with PM_residential heating_ were *r* = 0.17 and *r* = 0.06 for PM_exhaust_ and PM_wear_, respectively.

The total PM exposure levels, including regional background concentrations, have been presented previously^11^. Briefly, the total levels were relatively low by international comparisons in all cohorts, with annual levels of total PM_2.5_ mostly below the current Swedish regulations of 10 µg/m^3^ but above the WHO 2021 air quality guideline of 5 µg/m^3^ (mean cohort lag 1–5 exposures 6.9 µg/m^3^ for PM_2.5_, and 11.3 µg/m^3^ for PM_10_). Total PM exposures were the highest in the PPS and GM cohorts in Gothenburg (lag 1–5 year PM_2.5_ 9.3 and 9.0 µg/m^3^, respectively) and the lowest in the VIP cohort in Umeå (lag 1–5 year PM_2.5_ 5.8 µg/m^3^).

### Associations between exposures and mortality

**Fig. 3:**
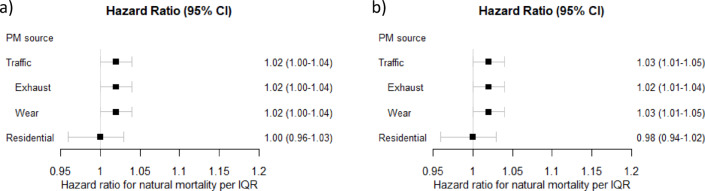
Meta-estimates of associations between natural mortality and (**a**) lag 1–5 and (**b**) lag 6–10 exposure to particulate matter (PM) air pollution from different sources, per interquartile ranges (IQRs), using the main covariate model. Hazard ratios (HR: s) and 95% confidence intervals (CI: s).

**Fig. 4:**
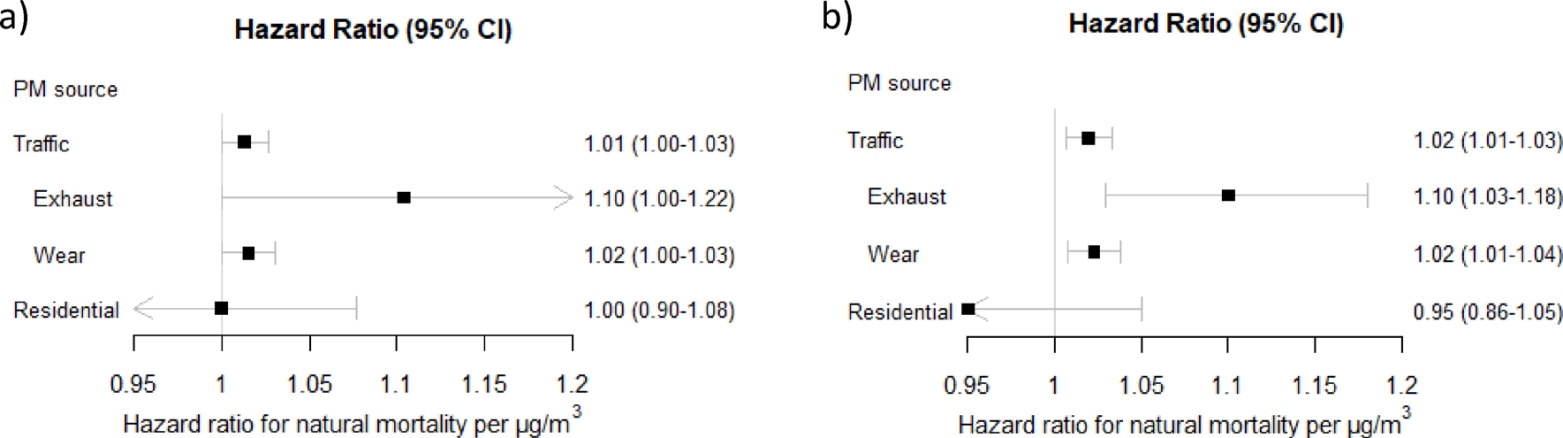
Meta-estimates of associations between natural mortality and (**a**) lag 1–5 and (**b**) lag 6–10 exposure to particulate matter (PM) air pollution from different sources, per 1 µg/m^3^, using the main covariate model. Hazard ratios (HR: s) and 95% confidence intervals (CI: s).

Residential exposure to PM_exhaust_ in the last five years (lag 1–5) was in the meta-regression associated with an increased risk of natural mortality (HR 1.02, 95% CI 1.00-1.04, per IQR of 0.2 µg/m^3^, HR 1.10, 95% CI 1.00-1.22, per 1 µg/m^3^, Figs. 3a and 4a). The association was similar for the highly correlated exposure to PM_wear_ (HR 1.02, 95% CI 1.00-1.04, per IQR of 1.3 µg/m^3^ and HR 1.02, 95% CI 1.00-1.03, per 1 µg/m^3^) and consequently also for combined PM_traffic_. The associations were overall comparable for exposure 6–10 years before death (Figs. 3b and 4b). In the cohort-specific analyses, the associations with traffic-related PM were overall positive in PPS and CEANS and null in GM, and in VIP null in lag 1–5 and positive in lag 6–10, but with wide confidence intervals (figures s2-s4). For PM_residential heating_ there was no association with increased natural mortality (HR 1.00 95% CI 0.96–1.03 per IQR of 0.4 µg/m^3^; HR 1.00 95% CI 0.90–1.08 per 1 µg/m^3^, and results were similar and non-significant in the separate cohorts (figure s5).

In multi-pollutant models, the associations between PM_exhaust_ and natural mortality were essentially unchanged by the inclusion of PM_residential heating_ in the model, and vice versa (figures s6-s7). Adjustment for residential road traffic noise (in the cohorts in Stockholm and Gothenburg where traffic noise could be assigned) did not affect the associations between PM_traffic_ and natural mortality (figure s8). There was no association between road traffic noise and natural mortality, when adjusted for PM_traffic_ (figure s9).

**Fig. 5:**
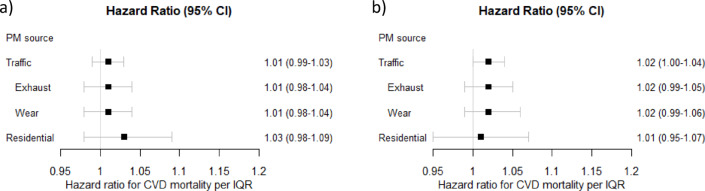
Meta-estimates of associations between cardiovascular (CVD) mortality and (**a**) lag 1–5 and (**b**) lag 6–10 exposure to particulate matter (PM) air pollution from different sources, per interquartile ranges (IQRs), using the main covariate model. Hazard ratios (HR: s) and 95% confidence intervals (CI: s).

**Fig. 6:**
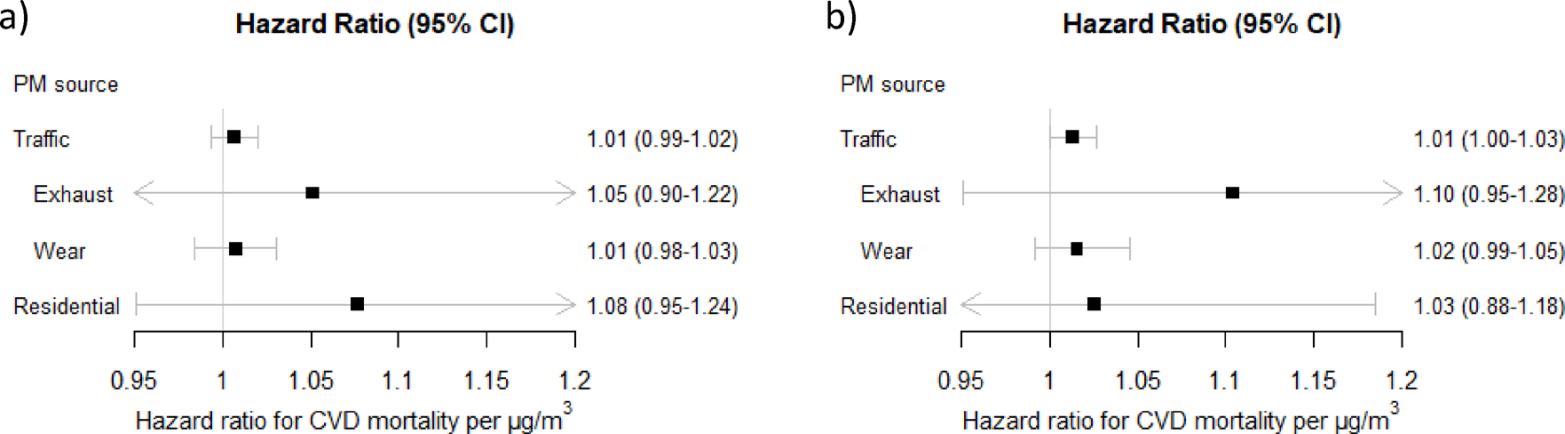
Meta-estimates of associations between cardiovascular (CVD) mortality and (**a**) lag 1–5 and (**b**) lag 6–10 exposure to particulate matter (PM) air pollution from different sources, per 1 µg/m^3^, using the main covariate model. Hazard ratios (HR: s) and 95% confidence intervals (CI: s).

Regarding CVD mortality, the associations with traffic-related PM were generally positive but weaker than for natural mortality and not statistically significant, for lag 1–5 and lag 6–10 (Figs. 5 and 6). For PM_residential heating_ there were also non-significant positive associations for lag 1–5, and null associations for lag 6–10. None of the specific cohorts had significant positive associations between traffic-related PM, or PM_residential heating_, and CVD mortality (figures s10-s13). Associations between PM_exhaust_ and CVD mortality were only marginally affected by adjusting the results for PM_residential heating_ (figure s14-15), as were the associations between PM_traffic_ and CVD mortality when adjusted for road traffic noise (figures s16-s17).

In a dataset restricted to only participants with exposure for both lag 1–5 and lag 6–10, the associations with mortality outcomes were comparable between these time windows and not markedly altered compared to those in the full dataset (figures s18-s21). For the crude covariate model, the associations were overall similar to those for the main model (table s1). Statistical tests of the proportional hazards assumption in relation to particles did not show any deviation from proportionality for neither total natural nor for CVD mortality in any of the cohorts. No statistically significant heterogeneity between cohort specific results were identified (figures s2-21).

## Discussion

In this Swedish multi-cohort exposed to relatively low air pollution exposure levels, traffic-related PM was associated with total natural mortality, but PM from residential heating was not. The associations were generally consistent across cohorts and time-windows of exposure (lag 1–5 years and lag 6–10 years before death had largely overlapping confidence intervals), and not influenced by co-exposure to road traffic noise, PM from residential heating, or individual risk factors. The hazard ratios per IQR were modest and of borderline significance however, reflecting the low exposure contrasts in these low-exposure settings. When expressed per fixed increment the estimated associations are consistent with recent multicohort studies^30^ and with meta-estimates for total PM_2.5_ exposure^7^. The associations with PM from traffic exhaust and traffic wear could not be separated due to high spatial correlations between these exposures. For CVD mortality, the associations with traffic-related air pollutants were positive but not statistically significant.

In the same cohorts we have previously reported associations between total concentrations of ambient PM_2.5_, PM_10_ and black carbon (BC) and mortality^11^. The most consistent associations were with total natural and CVD mortality for PM_10_, but there were significant positive associations also for BC and natural mortality, and PM_2.5_ and CVD mortality. The relative risk increases for total PM_10_ were relatively strong compared to most studies and roughly comparable to the estimates we now report for PM_wear_ (2% per 1 µg/m^3^), but substantially weaker per µg than our findings for PM_exhaust_ (corresponding to 10% per 1 µg/m^3^). It should be noted, however, that PM_exhaust_ consists largely of ultrafine particles (UFP) and the total mass is thus low even when particle numbers are high. Per IQR, the associations with mortality we report here for PM_traffic_ (and both components) are smaller than those for PM_2.5_ and PM_10_, but similar to those for BC. As reported previously, we also observed associations between BC and incidence of stroke, where two-pollutant models showed positive associations for BC from traffic-exhaust but not from residential heating^10^. For incidence of ischemic heart disease (IHD), however, the associations were positive primarily with PM from residential heating, in line with the slightly positive associations we now report for CVD mortality.

Previous literature on source-specific air pollution is varied regarding exposure assessment and categorization, with many studies focusing on chemical constituents rather than emissions sources. There are some publications of long-term exposure to air pollution from traffic and heating and mortality, however. Positive associations with natural mortality for PM_2.5_ related to coal burning and traffic, but not biomass burning, were found also in the US American Cancer Society (ACS) Cancer Prevention Study-II cohort^31^. Further analyses of specific IHD mortality indicated that the excess risk of traffic-related PM was due to the diesel component^32^. Similarly, a study of the US National Health Interview Survey (NHIS) cohort reported significant positive associations with cardiopulmonary mortality only for PM_2.5_ from vehicles and not from any other source^33^. In the European multi-cohort ELAPSE project, source-specific exposures for the year 2010 was apportioned using principal component analysis (PCA) in a longitudinal study of associations with mortality^30^. Similarly to our findings, the associations were positive for traffic-related air pollutants and of comparable size (e g HR 1.06; 95% CI: 1.04 and 1.08 per 2.86 µg/m^3^ increase for the traffic component, compared to our HR: s of 1.10 (95% CI 1.00-1.22) per 1 µg/m^3^ of road traffic exhaust particles and 1.02 (95% CI 1.00-1.03) per 1 µg/m^3^ of road wear particles. However, in contrast with our null estimates for natural mortality and PM from residential heating, they report significant positive associations also for biomass burning and agriculture, and CVD as well as natural mortality. For crustal/soil sources, which partially represent road dust, they reported essentially null associations in the multipollutant models. The source apportionment methods differed compared to our study however, in that it grouped air pollution from biomass burning and agriculture together in the PCA, and that road dust (similar to our road wear) may have been attributed to both the soil component and the traffic component. Positive associations with mortality for PM both from traffic emissions (tailpipe and non-tailpipe) and biomass burning were also reported in a study of the whole population in Massachusetts, with markedly stronger associations for CVD mortality than natural mortality^34^. In contrast, a study of source-specific air pollution in female teachers in California found no associations between PM from traffic exhaust and either natural or CVD mortality^35^. For IHD mortality, however, they observed significant positive associations with PM_2.5_ from traffic exhaust, while for PM_2.5_ from wood burning there were null associations for all types of mortality.

Most previous studies report associations between both air pollution and road traffic noise and CVD incidence and mortality^36^, and stronger associations between total PM_2.5_ and (overall and specific types of) CVD mortality than natural mortality^1^. We also observed this in our previous analysis in the same cohorts^11^. Furthermore, mechanistic studies and human exposure studies (mainly of combustion particles) indicate that cardiovascular morbidity and mortality are central effects of the main mechanisms through how particles affect human health: pulmonary and systemic inflammation, neuroendocrine activation, and direct effects on blood vessels of ultrafine particles translocated into the blood stream – leading to e g impaired endothelial function, arterial stiffness, atherosclerosis, increased coagulability, and arrhythmia^4,37,38^. We thus expected to find stronger estimates for CVD mortality than for natural mortality, but did not do so for either PM_traffic_ or PM_residential heating_. The estimates were however of similar magnitude, and the confidence intervals showed substantial overlap. When comparing per µg/m^3^, the HR: s we observe for PM_exhaust_ and natural mortality are higher than recent meta-estimates for total PM_2.5_ (e g HR 1.095, 95% CI 1.064–1.127) per 10 µg/m³ in Orellano 2024^1^, while for PM_wear_ and PM_traffic_ the estimates are of comparable size.

Several issues should be considered when comparing how components or sources of air pollution relate to health effects, both within studies and between studies. One key difficulty is that the types and sources of PM modelled differ substantially between studies. The methods for air pollution modelling also vary between studies. Associations with mortality can be substantially different depending on modelling techniques^39^. Further comparisons of how modelling methods affect results are warranted. Multiple studies using the same methods for exposure assessment for different populations would be valuable to resolve this issue. We argue that defining PM according to emission sources is a useful approach since most abatement policies target specific emission sources or sectors, and findings can be more directly translated into targeted preventive measures compared to data on chemical composition or total concentrations. We observed the most consistent pattern of associations across cohorts, exposure windows, and models for traffic-related PM, compared to PM from residential heating. However, given the modest effect sizes, correlations between PM components and potential differential exposure misclassification, these findings should be interpreted as suggestive rather than definitive evidence of differential toxicity.

Another key problem is that the spatial correlation between PM from traffic exhaust and non-exhaust emissions is high. A true association with health outcomes for either component would thus lead to a statistical association also for the other. For Sweden, the percentage of vehicles that use studded tires during winter is an important determinant for non-exhaust PM. The modeling in the present study took into account the downward trend in the use of studded tires as well as differences between cities, but a better spatial distribution of the presence of studded winter tires would increase the distinction between traffic exhaust and non-exhaust PM. Modelling strategies that consider meteorological variables such as road surface moisture, variations in road pavement and winter-time road maintenance (salting and sanding) would also improve the validity^40,41^. A high spatial correlation between long-term PM from traffic exhaust and non-exhaust is however inherent when the main source is the same – road traffic. To elucidate the relative toxicity of exhaust and wear particles, other types of studies are needed, such as human experimental chamber studies with specific and controlled exposures from different sources. Toxicological studies have so far indicated that particles related to combustion and traffic are toxic, but rarely compared the toxicity of traffic exhaust and non-exhaust directly^5,42^. The issue is important for estimations of the burden of disease of air pollution, as shown by the more than five times higher exposure-response per µg/m^3^ for PM_exhaust_ compared to PM_wear_ in our results. The answer to this question also largely determines the size of the health benefit of the ongoing electrification of the vehicle fleet, since electrification removes exhaust PM but not the PM_wear_.

There may also be true regional differences in how different air pollutants or sources relate to health outcomes. Both air pollution levels and source profiles for air pollutants differ between areas, as do other demographic characteristics and populations health and risk factors that may affect susceptibility, as well as differences in data sources, including how outcomes are defined, coded, and recorded, which may influence comparability and introduce bias. This limits generalizability between studies and highlights that studies in different parts of the world are needed, and it may be problematic to combine them in meta-analyses. Our study participants consist of mostly urban middle-aged and older residents in northern Europe. Results are thus not necessarily valid for other geographical settings and populations, or for vulnerable subgroups of the population. Taken together, the conclusions of multiple reviews stating that current evidence does not allow us to differentiate the health effects of different types and sources of air pollution with certainty still stand^5,43^. Our results add some additional support for traffic-related PM being harmful. As the WHO 2021 air quality guidelines are implemented into EU and national legislations the coming years, regulation specifically targeting local emissions from road traffic should be considered as they may protect public health better than guidelines using mass-based PM^44^.

One possible limitation is that the emission data regarding residential wood burning and shipping is less spatially detailed and accurate compared to that for road traffic. Thus, one possible reason for finding associations with mortality for traffic-related PM but not for PM from residential heating is the lower precision and spatial resolution of the latter, leading to non-differential misclassification. This would bias the estimate towards the null and an underestimation of a true association between PM_residential heating_ and mortality. However, in the VIP-cohort where we had more precise emission data for residential heating associations with mortality outcomes were similar as for the other cohorts and overall non-positive. Furthermore, for cardiovascular mortality the hazard ratios for PM_residential heating_ were comparable to those of traffic-related PM. As accurate exposure assessments of PM from residential heating remains a challenge^45^, further efforts to improve emission inventories regarding residential wood combustion are important so that future studies can disentangle the health effects of PM from different sources better. Another limitation is that some individual-level covariates were not collected uniformly across cohorts and some (e g alcohol consumption) were missing for entire cohorts, which could potentially lead to different levels of residual confounding between cohorts. Since we considered the risk of confounding higher if we restricted the covariates to only those available in all cohorts, we used cohort-specific adjustment sets based on best available variables, as is common in multicohort studies. Additional adjustment in the cohorts where the missing variables existed had negligible impact on effect estimates, suggesting that differential covariate availability is unlikely to explain our findings.

A strength of this study is that we were able to follow participants’ addresses longitudinally and assign individual residential exposure to source-specific ambient PM from high-resolution dispersion models with little missing data for a long study period. We also have cause-specific mortality data from high-quality registries, and detailed covariate data. Furthermore, adjustment for road traffic noise (a potentially important confounder, since traffic noise is correlated with traffic-related air pollutants and considered a major environmental risk factor for cardiovascular health), did not noticeably affect our finding. This is consistent with evidence indicating that air pollution and traffic noise may act through partially overlapping biological pathways, while remaining sufficiently independent environmental exposures to warrant joint consideration in epidemiological analyses^38^. While residual confounding can never be excluded, the risk is smaller in this study compared to those in large administrative cohorts without individual-level data. In previous analyses in the same datasets^10^, including or stratifying by cardiovascular risk factors did not affect the associations between air pollutants and ischemic heart disease and stroke, and it is unlikely that including these would have altered the associations for cardiovascular mortality to more positive associations. A limitation is that we could not assign exposure to other sources of air pollution than road traffic and residential heating with sufficient quality for separate analyses of associations with mortality. PM from shipping, industry and other sources had overall low concentrations, and the precision and level of detail for these sources were limited. Improved emission inventories for industrial emissions, shipping, off-road machinery and agricultural sources would be beneficial for exposure modelling and studies of health effects for PM from these sources. A limitation was also that road traffic noise was only available for the cohorts in Gothenburg and Stockholm and not the VIP cohort in Umeå. The overall low exposure levels and contrast in the study area may be considered a limitation as it limits statistical power, and interpretation of results with borderline statistical significance should be cautious as chance findings are possible. The low exposure levels are also a strength however as it enables us to study specifically the effects of low exposure concentrations in the general population. This makes the results particularly relevant for informing guidelines and long-term preventive action.

## Conclusions

In mostly urban population-based cohorts in Sweden with relatively low exposure levels, we found modest but statistically significant associations between long-term exposure to locally emitted traffic-related particulate air pollution and total natural mortality. While the estimated risk was low for each individual, the results indicate a need to decrease population exposure to air pollution from road traffic to protect public health, even in environments with already low exposure levels. In low-exposure settings, air quality guidelines and goals targeting local emissions from traffic should be considered. More and improved studies of the specific health effects of different types and sources of air pollutants are still warranted.

## Supplementary Information

Below is the link to the electronic supplementary material.

Supplementary Material 1

## Data Availability

The datasets used and analysed during the current study are available from the corresponding author on reasonable request. Approval from ethical review board will be required since it contains sensitive information.
