# Supplementary material for: Long-term exposure to particulate matter from road traffic and residential heating and mortality: a multi-cohort study in Sweden
Source: Sci Rep. 2026 Feb 27;16:7955. doi: 10.1038/s41598-026-37471-5 (PMC12957509; doi:10.1038/s41598-026-37471-5)
Supplement: Supplementary file 1 — Supplementary Material 1 [file 41598_2026_37471_MOESM1_ESM.docx]

## Appendix:


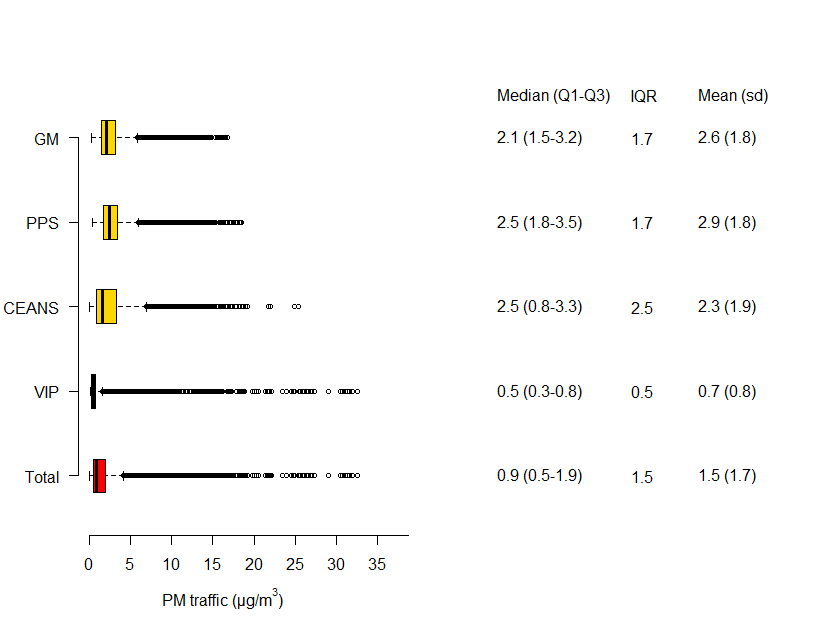


Supplemental fig s1: A boxplot of particulate matter (PM) traffic concentrations during the study period 1990-2011 in total and separate for the four cohorts. Tabulated values are quartiles (Q1=quartile 1, Q3=quartile 3), interquartile range (IQR), means and standard deviations (sd). GM=GOT-MONICA, PPS= Primary Prevention Study, CEANS= Cardiovascular Effects of Air pollution and Noise Study, VIP= Västerbotten Intervention Program.

Supplemental fig s2-s10: Natural mortality different sources

Supplemental fig s11-s17: Cardiovascular disease (CVD) mortality different sources

Supplemental figs s18-s21: Analyses between particulate matter (PM) and mortality in a data set restricted to only those with complete data for both lag1-5 and lag 6-10 exposure.


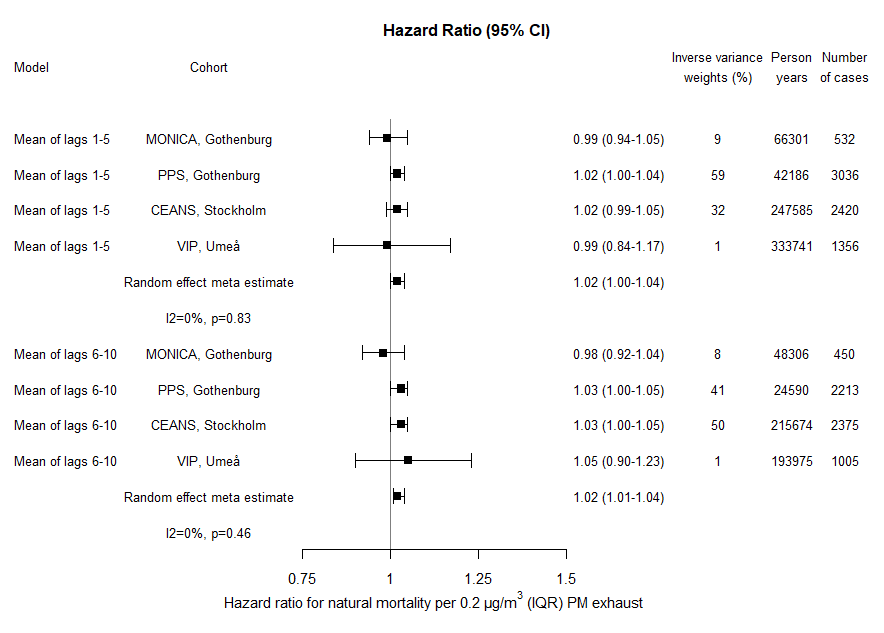
Supplemental Figure s2. Long-term exposure to traffic-exhaust particles (PM exhaust) and natural mortality in the four cohorts, per interquartile range (IQR) of 0.2 µg/m^3^, using the main covariate model. Hazard ratios (HR:s) and 95% confidence intervals (CI:s), weight, person-years and numbers of cases for lag 1-5 and lag 6-10. PPS= Primary Prevention Study, CEANS= Cardiovascular Effects of Air pollution and Noise Study, VIP= Västerbotten Intervention Program.


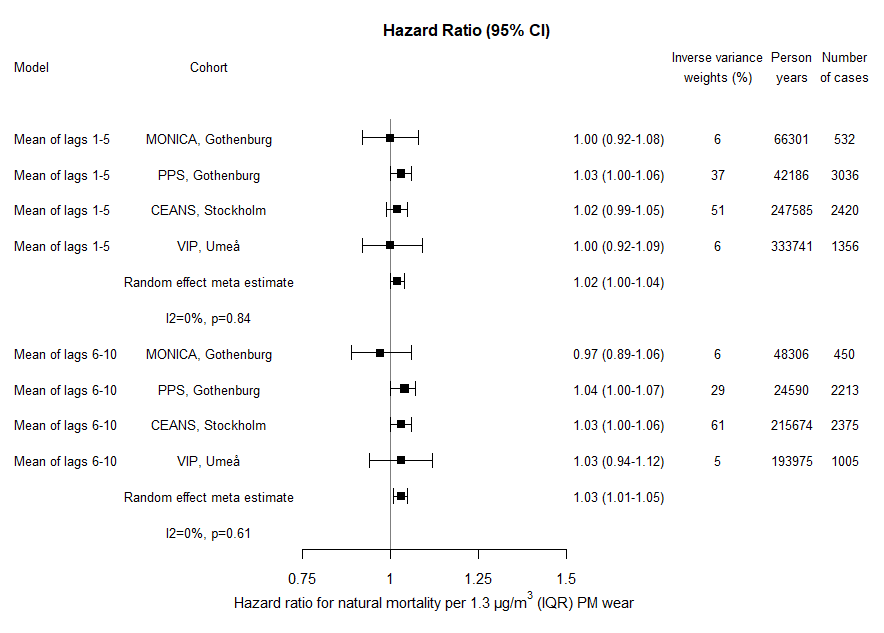


Supplemental Figure s3. Long-term exposure to traffic-wear particles (PM wear) and natural mortality in the four cohorts, per interquartile range (IQR) of 1.3 µg/m^3^, using the main covariate model. Hazard ratios (HR:s) and 95% confidence intervals (CI:s), weight, person-years and numbers of cases for lag 1-5 and lag 6-10. PPS= Primary Prevention Study, CEANS= Cardiovascular Effects of Air pollution and Noise Study, VIP= Västerbotten Intervention Program.


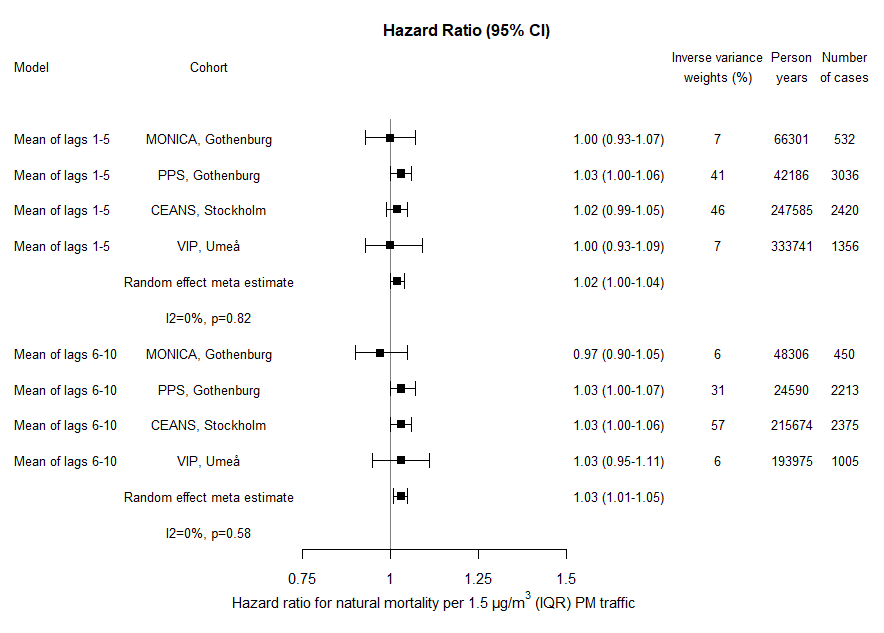


Supplemental Figure s4. Long-term exposure to total traffic-related particles (PM traffic) and natural mortality in the four cohorts, per interquartile range (IQR) of 1.5 µg/m^3^, using the main covariate model. Hazard ratios (HR:s) and 95% confidence intervals (CI:s), weight, person-years and numbers of cases for lag 1-5 and lag 6-10. PPS= Primary Prevention Study, CEANS= Cardiovascular Effects of Air pollution and Noise Study, VIP= Västerbotten Intervention Program.


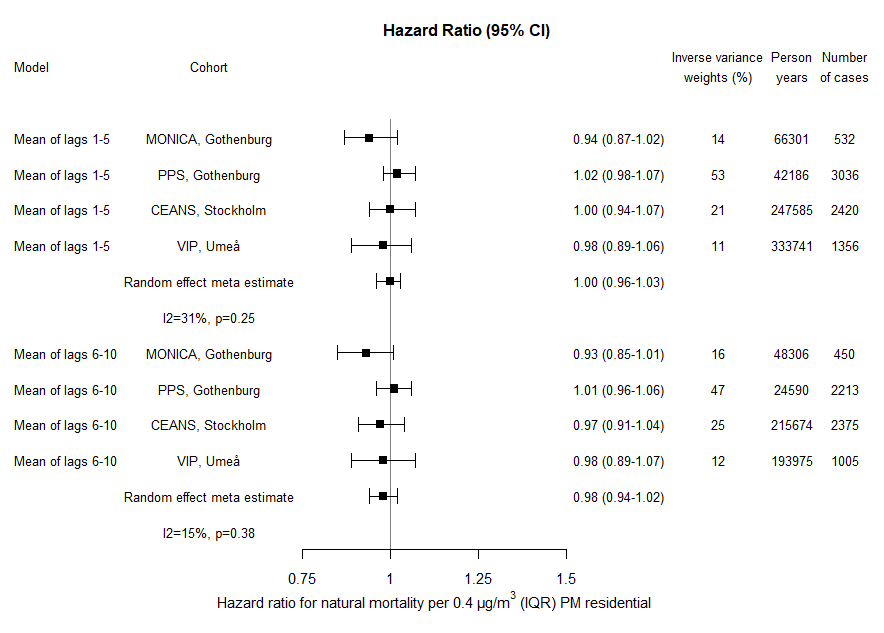


Supplemental Figure s5. Long-term exposure to particles from residential heating (PM residential) and natural mortality in the four cohorts, per interquartile range (IQR) of 0.4 µg/m^3^, using the main covariate model. Hazard ratios (HR:s) and 95% confidence intervals (CI:s), weight, person-years and numbers of cases for lag 1-5 and lag 6-10. PPS= Primary Prevention Study, CEANS= Cardiovascular Effects of Air pollution and Noise Study, VIP= Västerbotten Intervention Program.


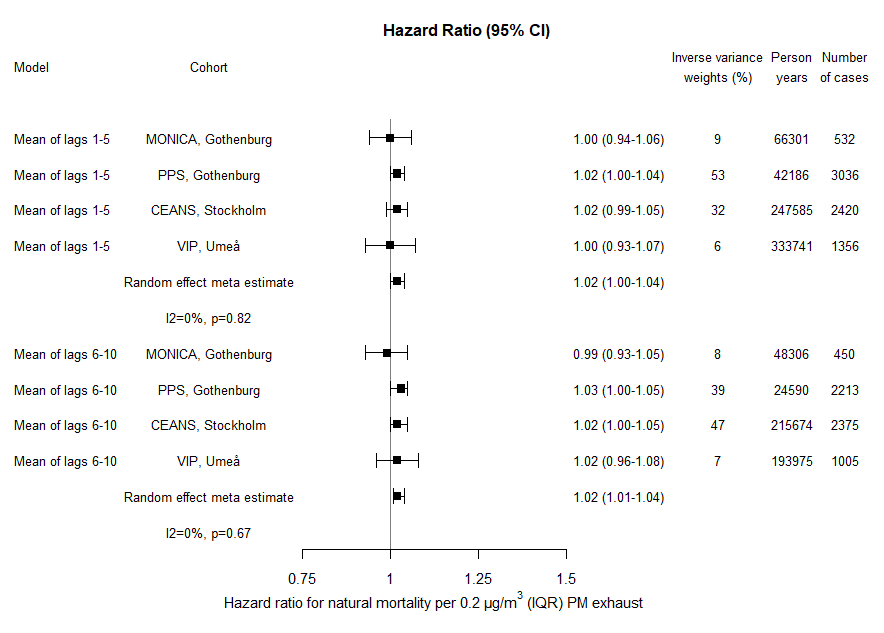


Supplemental Figure s6. Long-term exposure to traffic-exhaust particles (PM exhaust), adjusted for particles from residential heating, and natural mortality in the four cohorts, per interquartile range (IQR) of 0.2 µg/m^3^, using the main covariate model. Hazard ratios (HR:s) and 95% confidence intervals (CI:s), weight, person-years and numbers of cases for lag 1-5 and lag 6-10. PPS= Primary Prevention Study, CEANS= Cardiovascular Effects of Air pollution and Noise Study, VIP= Västerbotten Intervention Program.


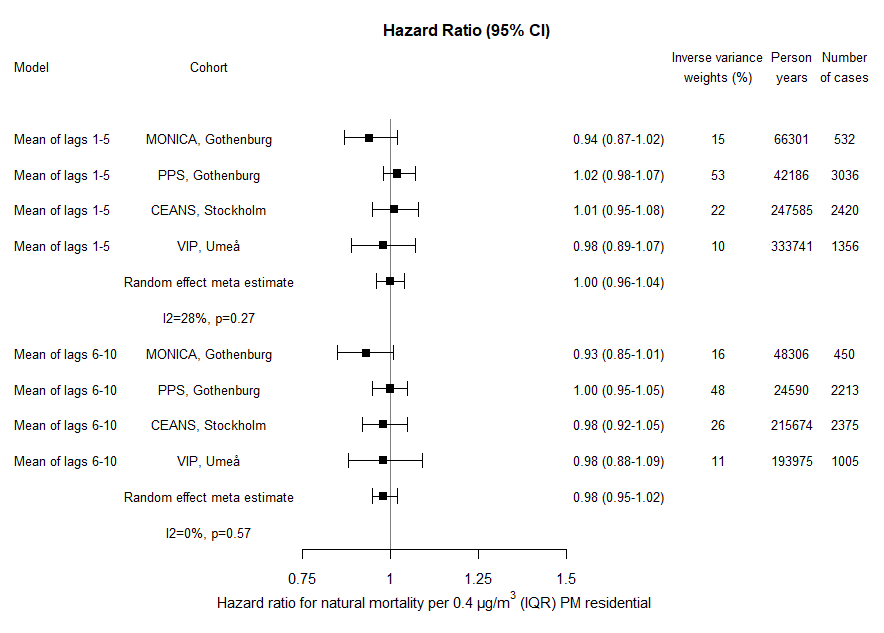


Supplemental Figure s7. Long-term exposure to particles from residential heating (PM residential), adjusted for traffic-exhaust particles, and natural mortality in the four cohorts, per interquartile range (IQR) of 0.4 µg/m^3^, using the main covariate model. Hazard ratios (HR:s) and 95% confidence intervals (CI:s), weight, person-years and numbers of cases for lag 1-5 and lag 6-10. PPS= Primary Prevention Study, CEANS= Cardiovascular Effects of Air pollution and Noise Study, VIP= Västerbotten Intervention Program.


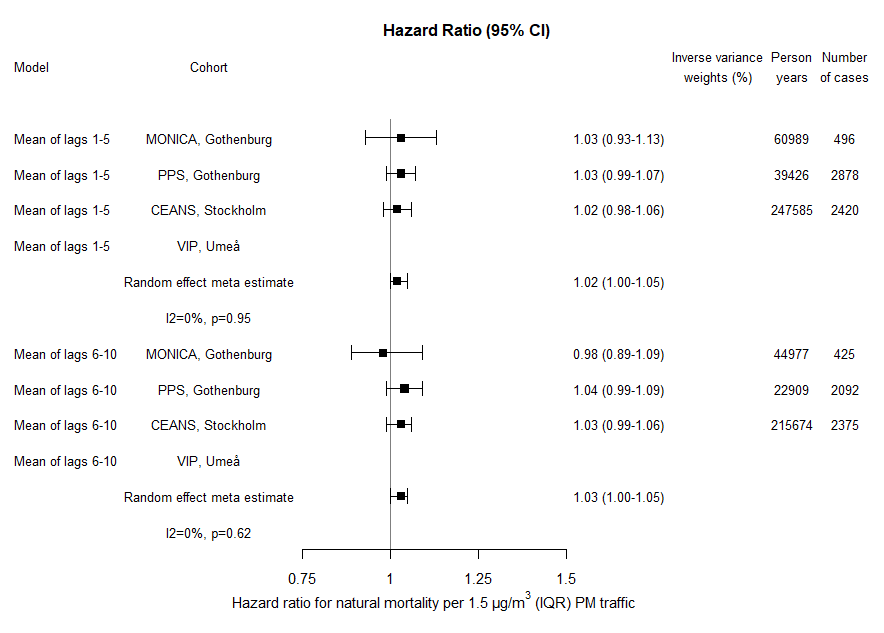


Supplemental Figure s8. Long-term exposure to total traffic-related particles (PM traffic), adjusted for road traffic noise, and natural mortality in the three cohorts where noise could be assigned, per interquartile range (IQR) of 1.5 µg/m^3^, using the main covariate model. Hazard ratios (HR:s) and 95% confidence intervals (CI:s), weight, person-years and numbers of cases for lag 1-5 and lag 6-10. PPS= Primary Prevention Study, CEANS= Cardiovascular Effects of Air pollution and Noise Study, VIP= Västerbotten Intervention Program.


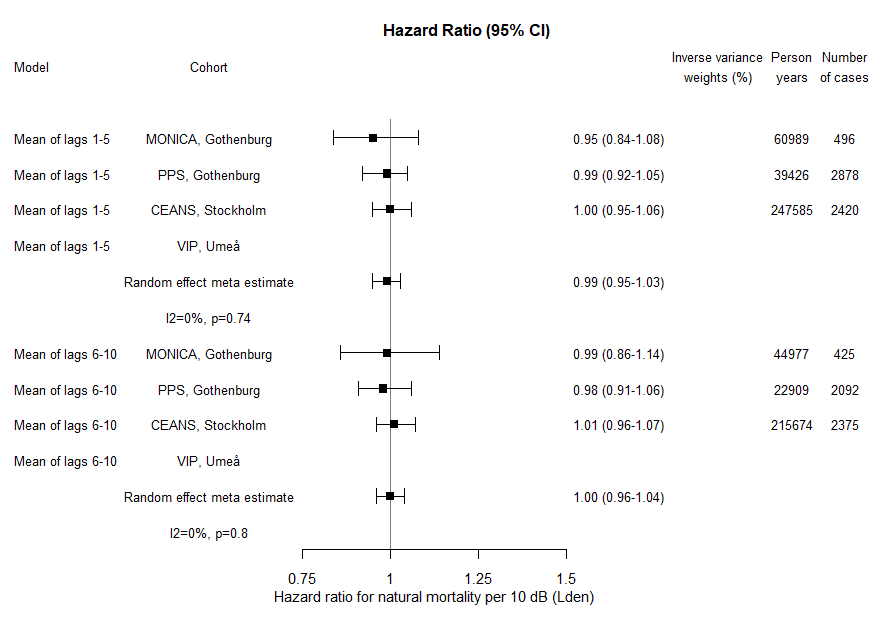


Supplemental Figure s9. Long-term exposure to road traffic noise and natural mortality in the three cohorts where noise could be assigned, per 10 dB (Lden) , using the main covariate model. Hazard ratios (HR:s) and 95% confidence intervals (CI:s), weight, person-years and numbers of cases for lag 1-5 and lag 6-10. PPS= Primary Prevention Study, CEANS= Cardiovascular Effects of Air pollution and Noise Study, VIP= Västerbotten Intervention Program.


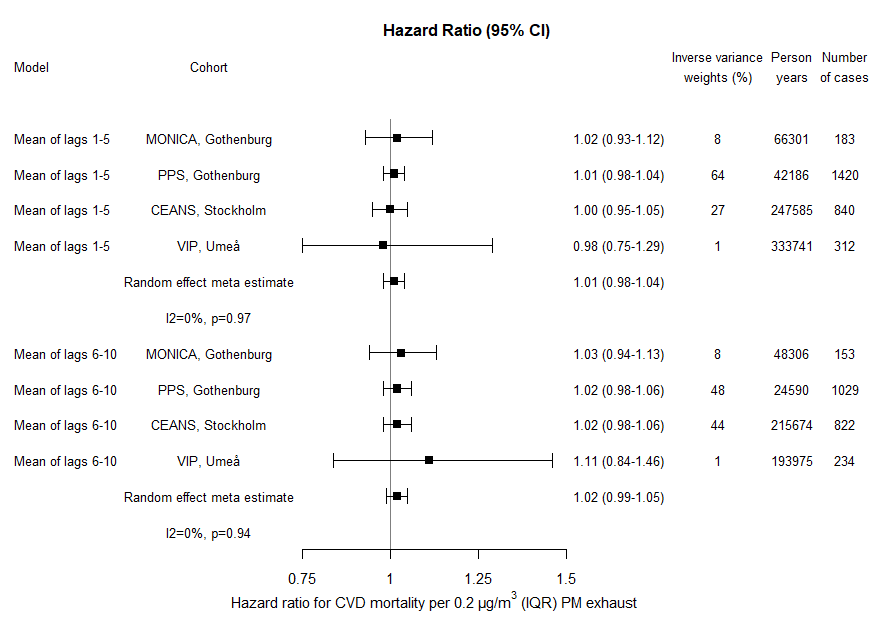


Supplemental Figure s10. Long-term exposure to traffic-exhaust particles (PM exhaust) and cardiovascular (CVD) mortality in the four cohorts, per interquartile range (IQR) of 0.2 µg/m^3^, using the main covariate model. Hazard ratios (HR:s) and 95% confidence intervals (CI:s), weight, person-years and numbers of cases for lag 1-5 and lag 6-10. PPS= Primary Prevention Study, CEANS= Cardiovascular Effects of Air pollution and Noise Study, VIP= Västerbotten Intervention Program.


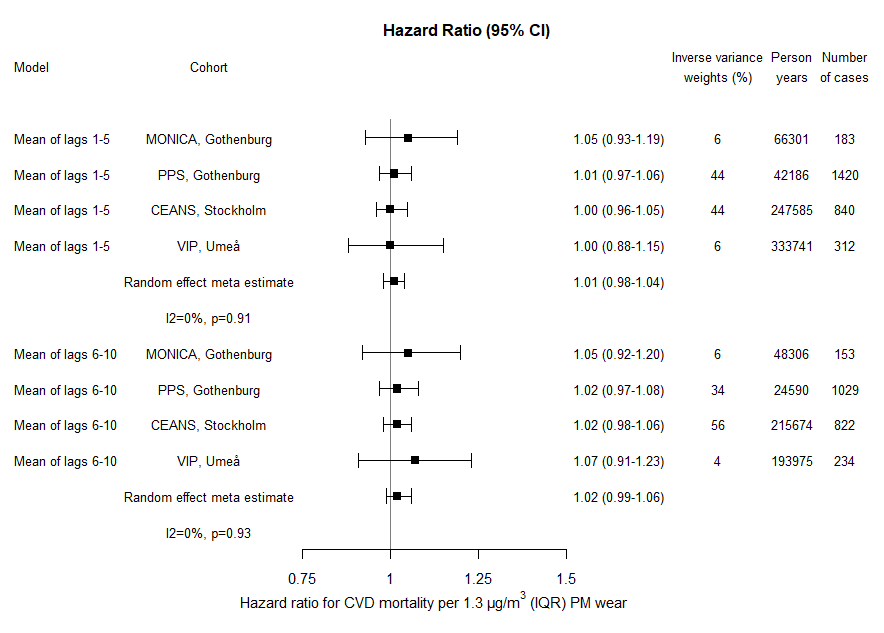


Supplemental Figure s11. Long-term exposure to traffic-wear particles (PM wear) and cardiovascular (CVD) mortality in the four cohorts, per interquartile range (IQR) of 1.3 µg/m^3^, using the main covariate model. Hazard ratios (HR:s) and 95% confidence intervals (CI:s), weight, person-years and numbers of cases for lag 1-5 and lag 6-10. PPS= Primary Prevention Study, CEANS= Cardiovascular Effects of Air pollution and Noise Study, VIP= Västerbotten Intervention Program.


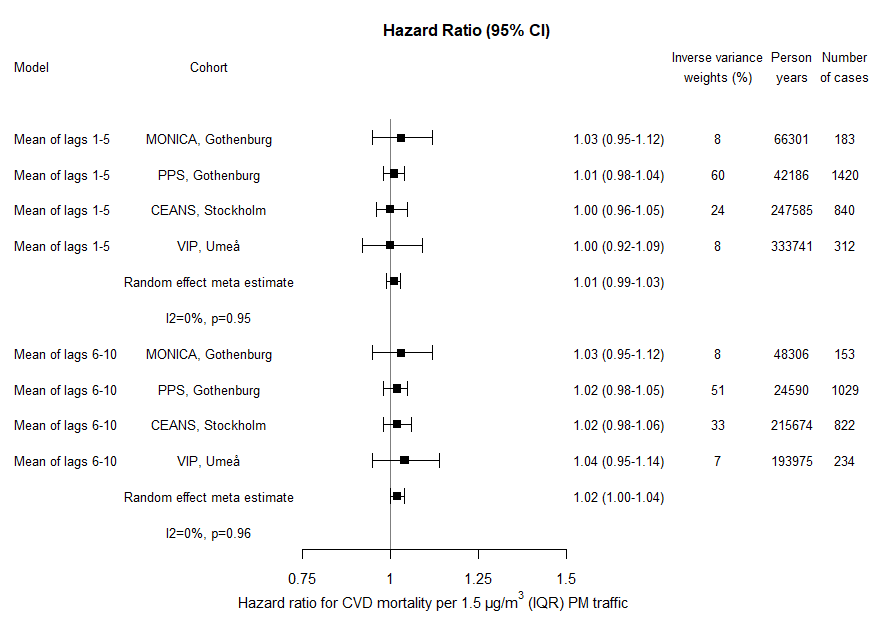


Supplemental Figure s12. Long-term exposure to total traffic-related particles (PM traffic) and cardiovascular (CVD) mortality in the four cohorts, per interquartile range (IQR) of 1.5 µg/m^3^, using the main covariate model. Hazard ratios (HR:s) and 95% confidence intervals (CI:s), weight, person-years and numbers of cases for lag 1-5 and lag 6-10. PPS= Primary Prevention Study, CEANS= Cardiovascular Effects of Air pollution and Noise Study, VIP= Västerbotten Intervention Program.


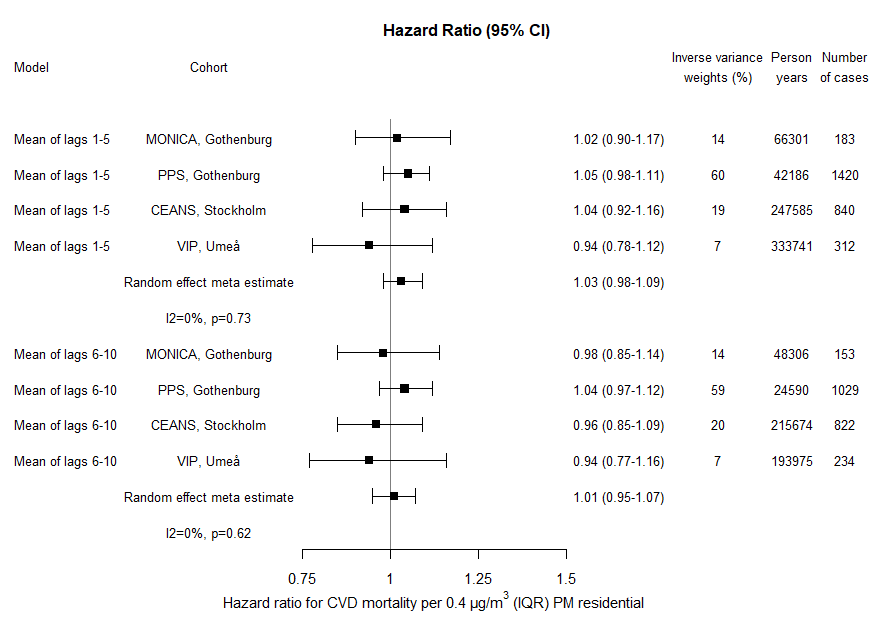


Supplemental Figure s13. Long-term exposure to particles from residential heating (PM residential) and cardiovascular (CVD) mortality in the four cohorts, per interquartile range (IQR) of 0.4 µg/m^3^, using the main covariate model. Hazard ratios (HR:s) and 95% confidence intervals (CI:s), weight, person-years and numbers of cases for lag 1-5 and lag 6-10. PPS= Primary Prevention Study, CEANS= Cardiovascular Effects of Air pollution and Noise Study, VIP= Västerbotten Intervention Program.


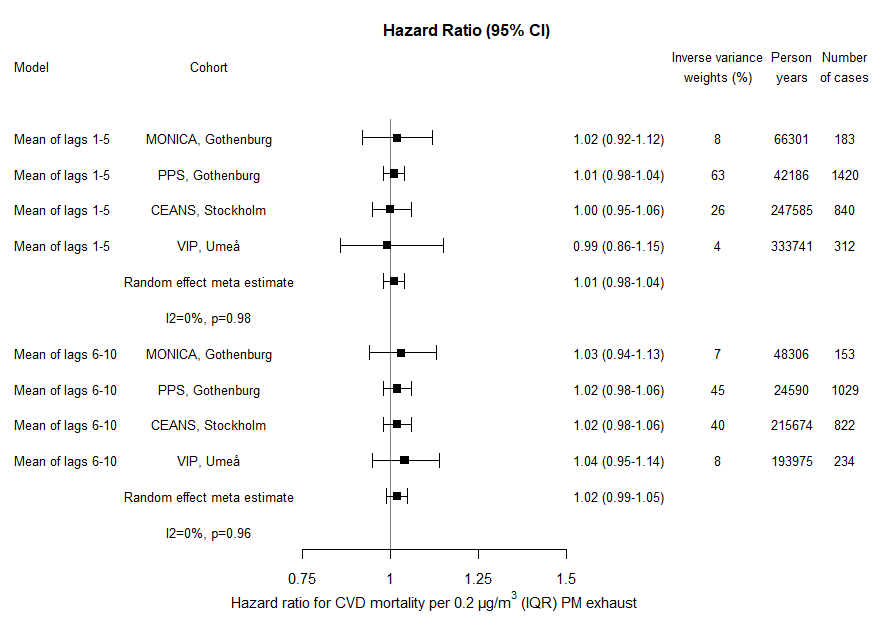


Supplemental Figure s14. Long-term exposure to traffic-exhaust particles (PM exhaust), adjusted for particles from residential heating (PM residential), and cardiovascular (CVD) mortality in the four cohorts, per interquartile range (IQR) of 0.2 µg/m^3^, using the main covariate model. Hazard ratios (HR:s) and 95% confidence intervals (CI:s), weight, person-years and numbers of cases for lag 1-5 and lag 6-10. PPS= Primary Prevention Study, CEANS= Cardiovascular Effects of Air pollution and Noise Study, VIP= Västerbotten Intervention Program.


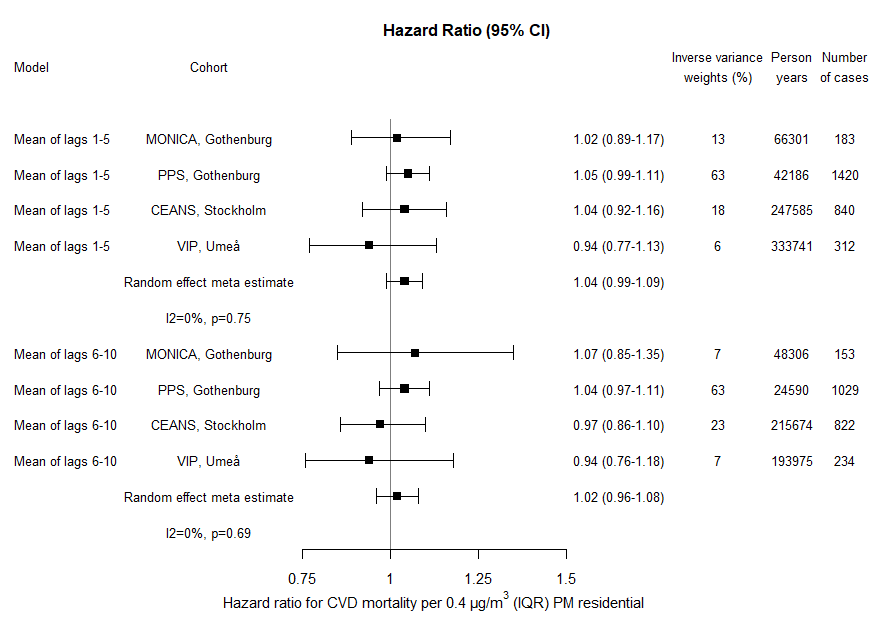


Supplemental Figure s15. Long-term exposure to particles residential heating (PM residential), adjusted for traffic-exhaust particles (PM exhaust), and cardiovascular (CVD) mortality in the four cohorts, per interquartile range (IQR) of 0.4 µg/m^3^, using the main covariate model. Hazard ratios (HR:s) and 95% confidence intervals (CI:s), weight, person-years and numbers of cases for lag 1-5 and lag 6-10. PPS= Primary Prevention Study, CEANS= Cardiovascular Effects of Air pollution and Noise Study, VIP= Västerbotten Intervention Program.


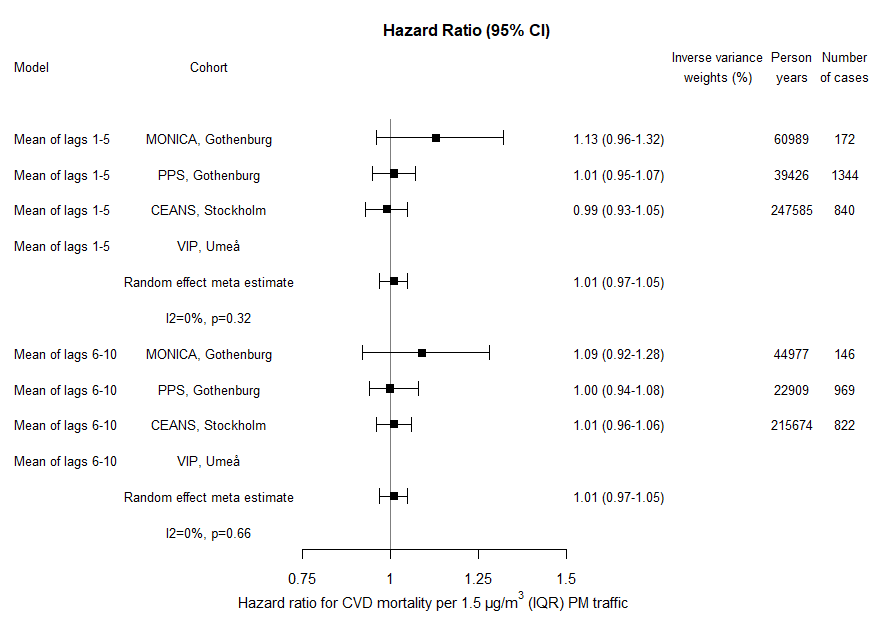


Supplemental Figure s16. Long-term exposure to traffic-related particles (PM traffic), adjusted for road traffic noise, and cardiovascular (CVD) mortality in the three cohorts where noise could be assigned, per interquartile range (IQR) of 1.5 µg/m^3^, using the main covariate model. Hazard ratios (HR:s) and 95% confidence intervals (CI:s), weight, person-years and numbers of cases for lag 1-5 and lag 6-10. PPS= Primary Prevention Study, CEANS= Cardiovascular Effects of Air pollution and Noise Study, VIP= Västerbotten Intervention Program.


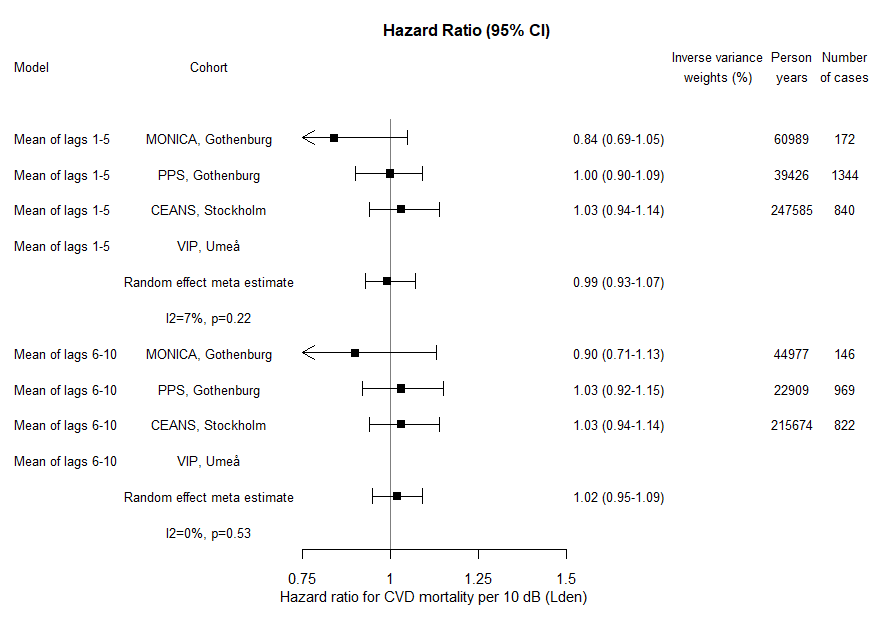


Supplemental Figure s17. Long-term exposure to road traffic noise and cardiovascular (CVD) mortality in the three cohorts where noise could be assigned, per 10 dB (Lden), using the main covariate model. Hazard ratios (HR:s) and 95% confidence intervals (CI:s), weight, person-years and numbers of cases for lag 1-5 and lag 6-10. PPS= Primary Prevention Study, CEANS= Cardiovascular Effects of Air pollution and Noise Study, VIP= Västerbotten Intervention Program.


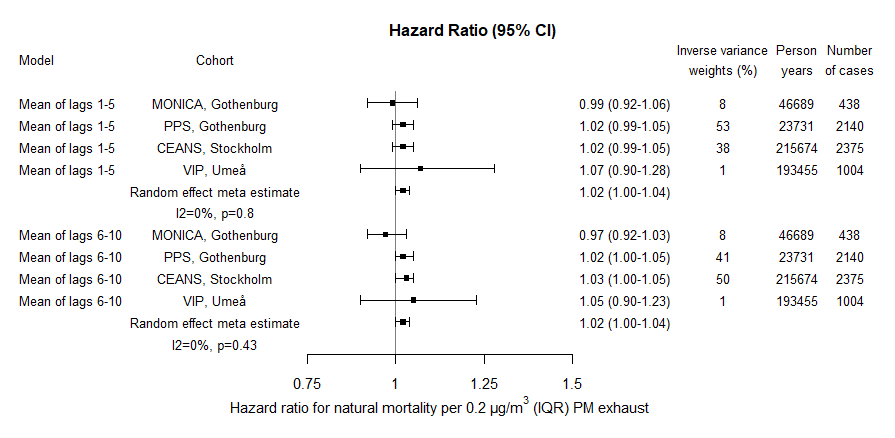


Supplemental Figure s18. Long-term exposure to traffic-exhaust particles (PM exhaust) and natural mortality in the four cohorts, per interquartile range (IQR) of 0.2 µg/m^3^, , using the main covariate model, with the data set restricted to only participants with exposure for both lag 1-5 and lag 6-10. Hazard ratios (HR:s) and 95% confidence intervals (CI:s), weight, person-years and numbers of cases for lag 1-5 and lag 6-10. PPS= Primary Prevention Study, CEANS= Cardiovascular Effects of Air pollution and Noise Study, VIP= Västerbotten Intervention Program.


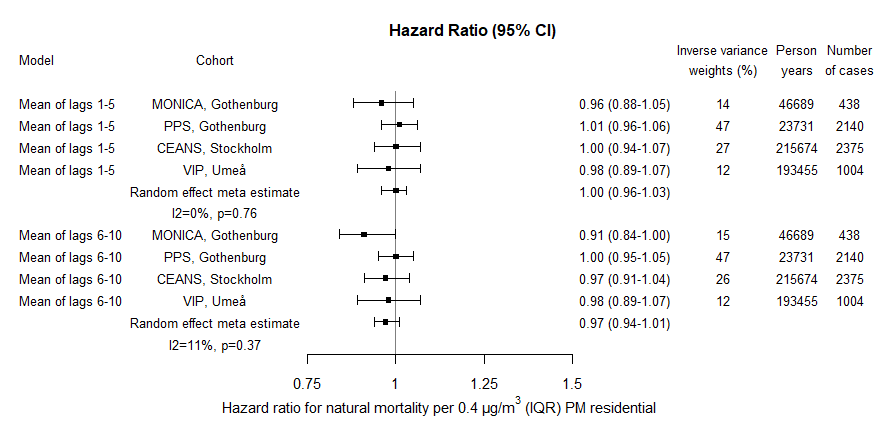
 Supplemental Figure s19. Long-term exposure to particles from residential heating (PM residential) and natural mortality in the four cohorts, per interquartile range (IQR) of 0.4 µg/m^3^, using the main covariate model, with the data set restricted to only participants with exposure for both lag 1-5 and lag 6-10. Hazard ratios (HR:s) and 95% confidence intervals (CI:s), weight, person-years and numbers of cases for lag 1-5 and lag 6-10. PPS= Primary Prevention Study, CEANS= Cardiovascular Effects of Air pollution and Noise Study, VIP= Västerbotten Intervention Program.


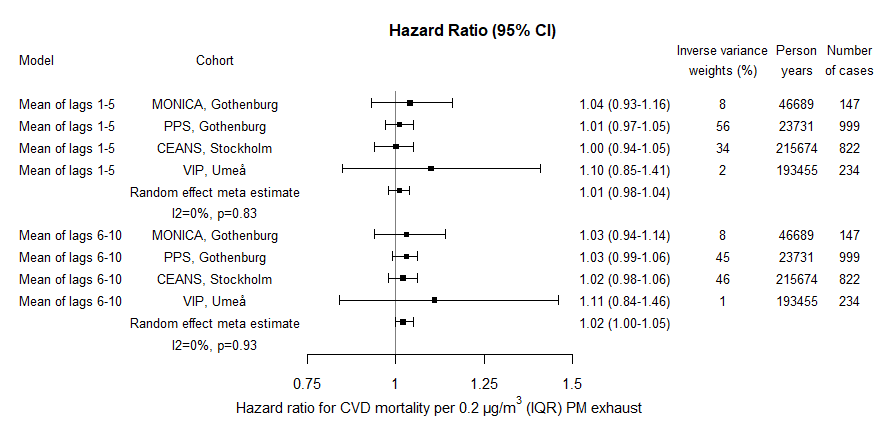


Supplemental Figure s20. Long-term exposure to traffic-exhaust particles (PM exhaust) and natural mortality in the four cohorts, per interquartile range (IQR) of 0.2 µg/m^3^, using the main covariate model, with the data set restricted to only participants with exposure for both lag 1-5 and lag 6-10. Hazard ratios (HR:s) and 95% confidence intervals (CI:s), weight, person-years and numbers of cases for lag 1-5 and lag 6-10. PPS= Primary Prevention Study, CEANS= Cardiovascular Effects of Air pollution and Noise Study, VIP= Västerbotten Intervention Program.
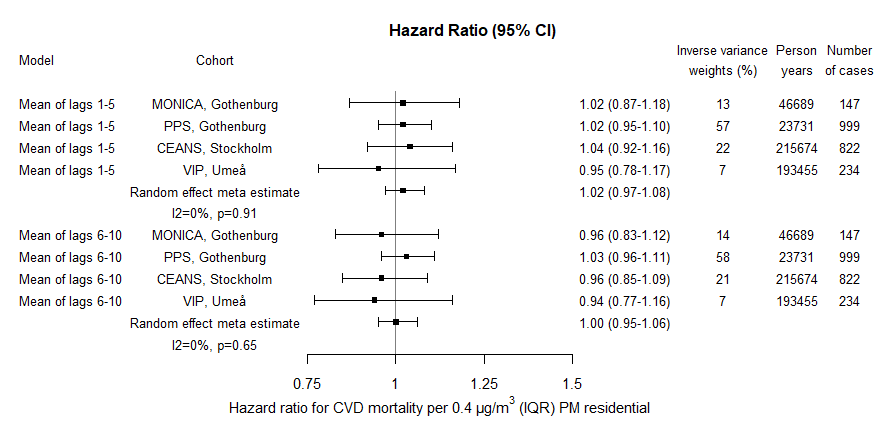


Supplemental Figure s21. Long-term exposure to particles from residential heating (PM residential) and cardiovascular (CVD) mortality in the four cohorts, per interquartile range (IQR) of 0.4 µg/m^3^, using the main covariate model, with the data set restricted to only participants with exposure for both lag 1-5 and lag 6-10. Hazard ratios (HR:s) and 95% confidence intervals (CI:s), weight, person-years and numbers of cases for lag 1-5 and lag 6-10. PPS= Primary Prevention Study, CEANS= Cardiovascular Effects of Air pollution and Noise Study, VIP= Västerbotten Intervention Program.

Table S1. Meta-estimates (Odds Ratios, OR) of associations between a) lag 1-5 and b) lag 6-10 exposure to particulate matter (PM) from different sources and natural and cardiovascular (CVD) mortality in the four cohorts, per interquartile range (IQR), in the crude covariate model adjusted only for sex, calendar year, and cohort (age set as timescale).

|  | OR for natural mortality | | OR for CVD mortality | |
| --- | --- | --- | --- | --- |
| PM source | lag 1-5 | lag 6-10 | lag 1-5 | lag 6-10 |
| Traffic | 1.02 (1.00-1.03) | 1.02 (1.01-1.04) | 1.01 (0.99-1.03) | 1.02 (1.00-1.04) |
| Exhaust | 1.04 (1.01-1.08) | 1.05 (1.02-1.09) | 1.04 (0.90-1.20) | 1.07 (0.98-1.18) |
| Wear | 1.01 (1.00-1.03) | 1.02 (1.00-1.03) | 1.01 (0.98-1.03) | 1.02 (1.00-1.05) |
| Residential heating | 0.94 (0.89-0.99) | 0.94 (0.89-0.99) | 1.00 (0.96-1.04) | 0.97 (0.91-1.03) |
